# Sex Differences in Pre-Season Anthropometric, Balance and Range-of-Motion Characteristics in Elite Youth Soccer Players

**DOI:** 10.3390/healthcare10050819

**Published:** 2022-04-28

**Authors:** Luis Llurda-Almuzara, Albert Pérez-Bellmunt, Noé Labata-Lezaun, Carlos López-de-Celis, Jason Moran, Nicholas C. Clark

**Affiliations:** 1 Departamento de Ciencias Básicas, Universitat Internacional de Catalunya, 08195 Sant Cugat, Barcelona, Spain; lllurda@uic.es (L.L.-A.); aperez@uic.es (A.P.-B.); nlabata@uic.es (N.L.-L.); 2 Departamento de Fisioterapia, Universitat Internacional de Catalunya, 08195 Sant Cugat, Barcelona, Spain; carlesldc@uic.es; 3 School of Sport, Rehabilitation, and Exercise Sciences, University of Essex, Wivenhoe Park, Colchester CO4 3SQ, Essex, UK; jmorana@essex.ac.uk

**Keywords:** youth, soccer, knee, ankle, injury, prevention, screening, anthropometric, balance, flexibility

## Abstract

In soccer, injury epidemiology differs between males and females. It is clinically useful to know whether there are between-sex differences in selected characteristics relevant to primary injury risk and injury prevention practices. The purpose of this study was to explore between-sex differences in anthropometric, balance, and range-of-motion characteristics in Spanish elite male and female youth soccer players. This was a pre-season cross-sectional study. Sixty-nine males (age 16.8 ± 0.9 yr; height 175.9 ± 6.8 cm; mass 67.9 ± 6.3 kg) and thirty-seven females (age 17.2 ± 1.7 yr; height 164.0 ± 6.3 cm; mass 59.0 ± 5.8 kg) participated. Anthropometrics (standing/sitting height, bodymass, right/left leg length) and right/left anterior reach test (ART), hip internal/external active range of motion, active knee extension (AKE), and weightbearing lunge test (WBLT) were measured. Between-sex differences were assessed with Bonferroni-corrected Mann–Whitney U tests and Cliff’s delta (d). Between-sex significant differences (*p* < 0.003, d ≥ 0.50) were observed for anthropometric data and for hip internal rotation. No between-sex significant differences were observed for ART/AKE/WBLT measures. Between-sex significant differences with large effect sizes were identified for anthropometric data and right/left hip internal rotation. The present study adds new data to the literature for young Spanish male and female soccer players. The present findings will help inform clinical reasoning processes and future injury prevention research for elite male and female youth soccer players.

## 1. Introduction

Soccer is one of the most popular sports in the world, with more than 270 million participants globally [1] and greater than 3.9 million children and adolescents playing annually [2]. Soccer is classed as a contact sport [3,4], within which players are required to perform physical tasks with high biomechanical impulses including sudden changes of direction, jumps, kicks, tackles, headers, and rapid accelerations and decelerations [5,6]. Because of the multi-directional and high biomechanical impulse nature of soccer, there is a substantial risk and high rate of lower-limb injuries across all competitive and age levels [7,8,9]. Youth soccer injury rates, specifically, have increased considerably in recent years [10,11]. In male and female youth soccer players, lower-limb injury rates of 5.70/1000 h of exposure and 6.77/1000 h of exposure, respectively, have been reported [12]. The thigh is most commonly injured in male youth soccer players whereas the knee is most commonly injured in female youth soccer players [12]. Specifically, males experience more frequent hamstring muscle strains than females (males 0.58/1000 athlete exposures; females 0.37/1000 athlete exposures) [13], whilst females experience more frequent anterior cruciate ligament (ACL) sprains than males (males 4.35/100,000 athlete exposures; females 13.23/100,000 athlete exposures) [14]. In general, females sustain more than twice the proportion of lower-limb joint injuries than males [12]. For male and female youth athletes, injuries result in profound consequences including physical disability [15,16], substantial health care costs [16], “negative psychological responses” [15], and academic study issues [15]. Therefore, because of the between-sex differences in injury frequency and the profound personal and socioeconomic consequences of injury, injury prevention interventions are needed to help mitigate the burden of injury for youth soccer players, their families, and wider society. Knowledge and understanding of between-sex differences in factors associated with injury risk are, subsequently, useful when considering the design of present-day or future generic and sex-specific lower-limb injury prevention interventions for youth soccer players.

Injury prevention programs aim to improve athletes’ risk factor profiles in order to reduce risk of injury [17,18,19]. Risk factors are characteristics that potentially increase the likelihood of sustaining an injury [20]. Modifiable risk factors (e.g., body mass) and nonmodifiable risk factors (e.g., height) can and cannot be altered with conservative interventions, respectively. Even though nonmodifiable risk factors cannot be altered with conservative interventions, it is useful to have knowledge and understanding of the presence and extent of nonmodifiable risk factors because they can affect the associations between, and interventions for, the remaining modifiable risk factors [21]. For example, in some youth soccer research, taller players have been reported to experience injury more frequently than their shorter peers [22] and heavier players have been observed to sustain injury more often than their lighter teammates [23]. Heavier limb segments and bodies generate higher forces and angular momentum [24,25]. Other work has reported that tibia length contributes to kinetics associated with noncontact knee injury [26]. Thus, in the context of noncontact injuries where there is no collision with an opponent [27], for players that are taller and heavier, interventions to modify body mass or the ability to better control body mass and biomechanical impulse may require emphasis given height and leg length (LL) cannot be altered. Further, in the context of contact injuries where there is collision with an opponent [27], such concepts extend to the design and implementation of youth mixed-sex training sessions and matches, which now occur frequently in soccer [28], because a mismatch between larger males and smaller females or smaller males and larger females may create a context in which contact injury risk is increased [29].

Anthropometrics including height [22,30] and body mass [23,31], dynamic balance performance [30,31,32], and lower-limb joint range-of-motion capability [31,33,34] have been reported as risk factors predisposing soccer players to lower-limb injury. Further, across a range of team sports, anthropometrics such as body mass have been associated with patellar tendinopathy [35] and plantar fasciitis [36], balance performance deficits have been associated with ankle sprains [37,38] as well as knee sprains and patellar tendinopathy [39], and range-of-motion deficits have been associated with injuries such as hamstring strain [40,41], ACL injury [42], and patellar tendinopathy [43]. Use of previous literature from a variety of study designs such as that just described can guide the design of preseason injury prevention screening procedures for agility-sport athletes [44]. Additionally, for valid clinical judgements to exist in injury prevention, exploratory research is also needed to understand biological phenomena and provide reference data from uninjured individuals [20]. Collectively, such research provides data to help reason whether injury prevention programs may need different components for male and female youth soccer players.

Exploratory research that provides reference data for uninjured youths should be culturally specific [45] and population specific [20]. Such reference data are important for informing current practice, building a foundation for the planning of future larger-scale projects, plotting a direction for new basic and applied research questions, and for cross-validating the data used in statistical analyses of future larger prospective studies [20]. To date, there is little exploratory research on anthropometric, dynamic balance, and range-of-motion characteristics for Spanish elite male and female youth soccer players. Therefore, the purpose of this study was to explore between-sex differences in anthropometric, dynamic balance, and range-of-motion characteristics in elite male and female youth soccer players in Spain using previously published injury prevention profiling procedures. It was hypothesized that there would be statistically significant between-sex differences for all characteristics. This exploratory analysis is original and practically significant because it provides new reference data that will inform present-day clinical reasoning and support the design and direction of future larger-scale primary studies examining lower-limb injury prevention in Spanish elite male and female youth soccer players.

## 2. Materials and Methods

### 2.1. Study Design and Setting

This was a cross-sectional study undertaken during the pre-season period. Data were collected in the first week prior to the 2021–2022 season. This study was part of a larger collaboration between the research team and local Spanish elite youth soccer clubs whose coaching and medical staff wished to engage in injury prevention screening for the welfare of their players; ‘elite’ players were defined as those playing at the highest Spanish football division accordingly to player’s age. Data were collected at the clubs’ training facilities in one session for each club. Because the clubs enrolled a set number of players for each season and wanted all players to be offered the opportunity to undertake injury prevention screening, a power analysis was redundant [46].

### 2.2. Ethical Approval, Participant Recruitment, Informed Consent

Ethical approval was obtained from the Institution Ethics Committee (CBAS-2021-06). Participants were recruited using flyers posted on noticeboards in open plan areas at club training facilities [46]. Participants aged ≥18 years signed an informed consent before participating in the injury prevention screening process and then agreeing to the use of their data for this study. For those aged under 18 years, informed assent was obtained along with informed consent from parents or legal guardians.

### 2.3. Participants

Inclusion criteria were: (1) to be a member of the Spanish Soccer Association, (2) to be an elite player (as defined above) and (3) to participate in at least four training and/or match sessions per week. Exclusion criteria were: (1) current time-loss injury in the previous two months before data collection, (2) to have trained within the two days before data collection, (3) to have had pain in any part of the lower limb at the time of data collection, and (4) any history of lower-limb surgery. One hundred and twelve soccer players volunteered to participate in this study, of which 106 (69 males, 37 females) met the eligibility criteria (Table 1). Finally, sixty-nine males (age from 15 to 19 yr; 51 players U18) and thirty-seven female (age from 15 to 20 yr, 25 players U18) youth soccer players from two clubs participated in this study.

### 2.4. Instrumentation

Standing and sitting height were measured with a conventional stadiometer (SECA, Barcelona, Spain). Body mass was measured with a conventional weighing scale (Bosch, Madrid, Spain). Leg length was measured with a conventional tape measure (TK Gruppe, Berlin, Germany). Hip rotation range of motion, hamstring flexibility, and ankle dorsiflexion range of motion were measured with a digital inclinometer (Fabrication Enterprises Inc., New York, NY, USA).

### 2.5. Procedures

Data collection was performed the day before pre-season training started. Data collection occurred at clubs’ medical services facilities. Players were instructed to avoid any fatiguing exercise or soccer training for 48 h beforehand.

Data collection procedures included, in order, dominant limb, height (standing, sitting), body mass, LL, the anterior reach test (ART), hip internal and external rotation range of motion, the active knee extension (AKE) test, and the weightbearing lunge test (WBLT). The order was chosen to minimize the effects of one procedure on the results of the next procedure. For LL, the ART, hip range of motion, the AKE test, and the WBLT, limb order was right then left.

#### 2.5.1. Dominant Limb

The dominant limb was defined as the self-reported preferred kicking limb [47].

#### 2.5.2. Height and Body Mass

Standing height was measured in centimeters (cm) to the nearest full cm using routine procedures [48]. For sitting height, also measured in cm, a 45 cm chair was used and participants were asked to stick the bottom to the end of the chair and keep the trunk upright [49,50]. Body mass was measured in kilograms (kg) using a conventional weighing scale [48]. Participants wore training clothes (shorts and short t-shirt) at the time of measurement. Standing and sitting height and body mass were performed barefoot.

#### 2.5.3. Leg Length

Participants were asked to lie in supine position on a stretcher. The measurement was taken in millimeters (mm) from the anterior superior iliac spine to the lower edge of the medial malleolus of the ankle [51]. Leg length assessment was performed barefoot. Reliability for this procedure has been reported (intraclass correlation coefficient (ICC) = 0.99) [51].

#### 2.5.4. Anterior Reach Test

Dynamic balance was measured using the ART [52]. In order to perform the ART, an inverted letter T was drawn on the ground. The roof of the letter T was considered to be the starting line. Participants were asked to stand on the test-leg with the tip of the first toe touching the posterior edge of the starting line. Participant’s hands had to be on the iliac crests during the procedure. They were asked to reach forward with the opposite foot and to touch the ground with the first toe as far as possible without lifting the heel of the supporting leg. The distance between the posterior edge of the starting line and the touching point was measured in cm to the nearest full cm using a traditional measuring tape. Participants were asked to perform three practice trials to neutralize acute learning effects and stabilize tissue hysteresis that could affect test values [53]. Subsequently, three measurements were performed and the maximum distance were used for statistical analysis. Participants performed all trials barefoot. Losing balance, removing the hands from the iliac crests, lifting the heel of the supporting leg, or not returning to the initial position invalidated the test. Reliability for this procedure has been reported (ICC = 0.95, standard error measurement (SEM) = 1.77 cm) [52].

#### 2.5.5. Hip Rotation Active Range of Motion

To assess hip rotation active range of motion, participants were asked to lie in prone position on a stretcher. In this position, the hip was maintained at 0° of extension and 20–30° of abduction as determined by researcher observation. The knee was flexed 90°. The pelvis was locked against the stretcher by means of a strap. From this position, participants were asked to rotate the hip as much as possible towards internal and external direction. The angle between the vertical and the tibia was measured in degrees (°) to the nearest full degree with the inclinometer. The inclinometer was placed on the distal part of the tibia; medial aspect 2 cm above medial malleollus for internal rotation and lateral aspect 2 cm above lateral maleollus for external rotation. A researcher monitored sacral tilt. Five external and internal hip rotations were performed to neutralize acute learning effects and stabilize tissue hysteresis that could affect test values [53]. Subsequently, three measurements were performed to evaluate internal hip rotation and three measurements to evaluate external hip rotation. The maximum angle between three tests were used for the statistical analysis. Participants performed all trials barefoot.

#### 2.5.6. Active Knee Extension Test

To assess hamstring flexibility, the AKE test was used [54]. Participants were asked to lie in supine position on the stretcher. A wooden box was used to maintain 90° of hip flexion during the procedure. The opposite limb was fixed against the stretcher by means of a strap. From this position, participants were asked to extend the knee as much as possible (maintaining the contact with the wooden box in order to maintain 90° of hip flexion). The angle between the vertical and the tibia was measured in degrees (°) to the nearest full degree using the inclinometer. Five maximal knee extensions were performed to neutralize acute learning effects and stabilize tissue hysteresis that could affect test values [53]. Subsequently, three measurements were performed and the maximal value was used for the statistical analysis. The inclinometer was placed on the tibial tuberosity for all the measurements. Participants performed all trials barefoot. The procedure was performed for both legs. Reliability for this procedure has been reported (ICC = 0.93; SEM = 4°) [54].

#### 2.5.7. Weightbearing Lunge Test

To assess ankle dorsiflexion range of motion, participants performed the WBLT [55]. The test consists of the patient standing in a tandem position and performing a forward stride. During this task, the foot involved remains firmly planted on the ground while the tibia advances over the talus to maximum ankle dorsiflexion. The angle between vertical and tibia was measured in degrees (°) to the nearest full degree using the inclinometer. The inclinometer was placed on the tibial tuberosity of the tested leg. Three maximal ankle dorsiflexion movements were performed to neutralize acute learning effects and stabilize tissue hysteresis that could affect test values [53]. Subsequently, three measurements were performed and the maximal angle used for the statistical analysis. Participants performed all trials barefoot. The procedure was performed for both legs. Reliability for this procedure has been reported (ICC = 0.96; SEM = 1.4°) [55].

### 2.6. Statistical Analysis

There were no missing data. Leg length measurements were converted from mm to cm to one decimal point. All ART trials were normalized by LL following Clark et al. [56]: percent leg length (%LL) = (reach distance (cm)/leg length (cm)) × 100. Both normalized and non-normalized ART scores were used for statistical analysis. All statistical analysis were performed using the Jamovi program (Jamovi software, Sydney, Australia) [57]. Descriptive statistics were calculated. Normality of data was assessed using visual inspection of histograms and Shapiro–Wilk tests. Alpha level was set a priori at 0.05. More than half of variables were not normally distributed and, therefore, nonparametric methods were used for all between-group comparisons. Differences between sexes were tested with the Mann–Whitney U test. Alpha level was again set a priori at 0.05 and Bonferroni correction was used for multiple comparisons (0.05/17) [20]. No sensitivity analyses were performed. Cliff’s delta was used for effect size estimates [58]; values of 0.14, 0.33 and 0.47 were interpreted as small, medium and large, respectively [58,59].

## 3. Results

Descriptive statistics are presented in Table 1 and Table 2. Eighty-four percent of the sample were right legged and 16% were left legged. No participant experienced pain during data collection. There were no adverse events.

Significant between-sex differences (*p* < 0.003) were found for all anthropometric data (Table 1). Large effect sizes were evident for standing and sitting height, body mass, and both left and right LL (Table 1). Anterior reach test data alone and normalized by LL showed no significant between-sex differences for the right or left limb (Table 2). Hip internal rotation was significantly different between sexes (*p* < 0.003) for both right and left limbs with large effect sizes evident (Table 2). However, this difference was not found for hip external rotation (Table 2). No significant between-sex differences were found for the AKE test or WBLT for either limb (Table 2).

## 4. Discussion

This study analyzed between-sex differences in anthropometric, balance, and range-of-motion characteristics in elite male and female youth soccer players in Spain. It was hypothesized that there would be statistically significant between-sex differences for all characteristics. Between-sex significant differences were only identified for anthropometric and hip internal rotation range-of-motion data.

Sex differences in height and body mass are negligible prior to puberty and the adolescent growth spurt [60]. Generally, standing height and body mass show between-sex divergence in the early teenage years although the velocity of growth and change in each begins to accelerate prior to age 13 years [60]. Girls tend to show acceleration in growth prior to boys at around age 10 years; after which, girls’ and boys’ standing heights tend to peak at approximately age 16 and 19 years, respectively [60]. Both girls’ and boys’ body mass can tend to increase into early adulthood and beyond [60]. Our findings demonstrate significant between-sex differences with large effect sizes for all anthropometric measures (Table 1). These findings are not surprising and are consistent with what would be expected for male and female soccer players of the mean and median ages observed in this study [60,61,62]. Few authors, however, have reported the mean or median LL in male and female adolescents and young adults [63,64]. The present study adds new data to the literature regarding mean and median LL specifically in young male and female soccer players. Observation and understanding of between-sex differences in anthropometry are important for normalization of data in studies that perform between-sex comparisons in physical ability [65], estimate injury risk [66] or conduct bio-banding in sport [67]. Data on between-sex differences in anthropometry are, therefore, important for informing reasoning and decision making across multiple aspects of soccer participation with young male and female players. For example, for those involved in reasoning a club’s programming strategies or decision making for a club’s operational policies, between-sex differences in body mass can be influential for informing the design and implementation of mixed-sex training sessions and matches [29].

There were no between-sex significant differences either for ART distance or ART normalized by LL (Table 2). These findings are consistent with previous work which also reported no between-sex differences for ART performance [68,69,70]. A recent systematic review and meta-analysis also concluded there were generally no between-sex differences for ART performance [71]. In contrast, other studies with similar sample characteristics to the current work report significant between-sex differences for the ART were present for soccer players [72] and basketball, volleyball, and tennis players [73]. Thus, more studies with larger samples are needed to draw firm conclusions about whether between-sex differences exist for dynamic balance defined by the ART. Given ART performance is associated prospectively with lower-limb injury risk [74], further data on the presence or absence of between-sex differences in dynamic balance would inform whether dynamic balance interventions should be considered for injury prevention programs with youth male or female soccer players, or both.

We found between-sex significant differences in hip maximum internal rotation but not in hip maximum external rotation (Table 2). Maximum external rotation was higher than internal rotation in both males and females (Table 2). Other studies also report between-sex significant differences with greater internal rotation but not external rotation range of motion for females compared to males in young soccer players [75], tennis players [76], and university multi-sport athletes [77]. The differences between males and females for hip internal rotation are likely multi-factorial. One factor is that females consistently demonstrate greater hip anteversion than males across the lifespan [78,79,80], which manifests clinically as greater hip maximum internal rotation range of motion [79]. Another factor is that bone strain due to hip muscle activity is thought to influence femoral modeling [81]. Given that males are consistently stronger in the hip muscles than females [82,83], it may be that between-sex differences in hip muscle activation and strength influence femoral and hip joint bone modeling in a way that then influences hip internal rotation range of motion. Therefore, between-sex differences in hip internal range of motion should be interpreted with care given female players likely possess different hip joint bony and capsular anatomy to males. Such interpretations will inform clinical reasoning with regard to whether an individual male or female player can be deemed to have a ‘stiff’ or ‘loose’ hip joint; this will then, in turn, inform clinical decision making for whether an individual player undertakes joint ‘mobility’ or ‘stability’ exercises as part of a pre-season injury prevention program [46], or whether no intervention is required at all.

Regarding hamstring flexibility, this study did not observe between-sex significant differences (Table 2). Other studies have measured hamstring flexibility using AKE test with mean values ranging from 19.9 to 38.0° for males [84,85,86,87,88] and 16.8 to 31.0° for females [84,85,86,88]. Our findings (Table 2), subsequently, appear consistent with the range of values reported in the literature and indicate between-sex differences in hamstring flexibility defined by the AKE test do not generally exist. One study, however, observed between-sex significant differences with males extending less than females [89]; the explanation for the different findings between their study and ours may lie in the characteristics of the sample, as Schulze et al. carried out the study on a sample of fitness athletes. Therefore, more research is necessary to confirm whether between-sex differences for hamstring flexibility measured by AKE test do exist specifically in elite youth soccer players.

In elite adult soccer players, ankle dorsiflexion range of motion is of interest from primary and secondary injury prevention and performance optimization perspectives [46]. We did not observe between-sex significant differences for maximum ankle dorsiflexion as represented by the WBLT in the present sample of elite youth soccer players (Table 2). Onate et al. [90] found between-sex differences for ankle dorsiflexion range of motion but grouped left and right limbs together for analysis and measured the ankle dorsiflexion capability in centimeters versus degrees. Consistent with our findings, Miler et al. [91] did not identify between-sex significant differences for ankle dorsiflexion range of motion in gymnasts. Other studies [92,93,94] have found mean weightbearing ankle dorsiflexion values higher than ours, ranging from 45.1° to 50.4° without comparing by sex. However, none of the studies employed a soccer player sample suggesting that soccer players could have lower weightbearing ankle dorsiflexion compared to other sports. Limited ankle dorsiflexion range of motion is associated with dynamic knee valgus, anterior knee pain, patellar tendinopathy, chronic ankle instability, Achilles’ tendinopathy, and metatarsal stress fractures [95]. Taking into account results from this study and previous literature, male and female elite youth soccer players may need to consider improving ankle dorsiflexion range of motion to decrease the risk for a variety of knee, ankle, and foot injuries.

Some potential limitations of this study include not performing an a priori power analysis and that approximately twice as many males were recruited compared to females. This was because the present study engaged with only two clubs with each club enrolling a set number of male and female players each season from whom participants could be recruited. In such a context, a priori power analyses are redundant [46] and between-group comparisons using variables with different distributions and variances can be performed using nonparametric statistics and effect size estimates [20,68]. Potential limitations also include performing right versus left leg analyses rather than dominant versus nondominant leg analyses. We did not undertake dominant versus nondominant leg analyses because dominance changes according to the nature of the task [96]. Accordingly, it is unclear precisely why some individuals choose one limb to perform a task and other individuals choose the opposite limb to perform the same task [97], and because task-specific side preference in unilaterally oriented sports may not yield anatomical and motor performance adaptations that are consistent with said side preference [56]. Therefore, right versus left leg analysis rather than dominant versus nondominant leg analysis is an acceptable procedure in studies including side-to-side comparisons [46,56,68]. Potential limitations further include performing hip internal and external rotation range-of-motion measurements in 20° versus 0° hip abduction. A starting position of 20° hip abduction was used to increase congruence of the hip articular surfaces and reduce ‘starting’ tension in the hip capsule and ligaments [98]. The starting position, however, was a limitation because the reliability and validity of such a procedure has not yet been established and because most previous research employs 0° abduction which affects the ability to compare and contrast our findings with that of others.

This study’s findings are only generalizable to similar samples of elite youth soccer players. Future research should compare male and female groups of similar sizes from a larger number of teams. Future research may also consider contrasting the findings of right versus left leg analyses with dominant versus nondominant leg analyses to determine if such different analyses actually yield different findings. Future research should further establish the reliability and validity of new clinical methods to assess hip internal and external range of motion. Researchers can use the findings of this study for plotting new directions for future basic and applied research questions and for cross-validating the data used in statistical analyses of future prospective studies in lower-limb injury prevention in elite male and female youth soccer players.

## 5. Conclusions

The data collection methods used in this study were safely employed with elite male and female youth soccer players. Between-sex significant differences with large effect sizes were identified for all anthropometric data and right and left hip maximum internal rotation. The present study adds new data to the literature regarding mean and median LL specifically in young male and female soccer players. No significant between-sex differences were identified for dynamic balance, hamstring flexibility, or ankle dorsiflexion range of motion as represented by the ART, AKE test, and WBLT, respectively. Between-sex differences in hip internal range of motion should be interpreted with care given female players likely possess different hip joint bony and capsular anatomy to males. When comparing the findings of this study to previous literature, male and female elite youth soccer players may need to consider improving ankle dorsiflexion range of motion to decrease the risk for a variety of knee, ankle, and foot injuries. The findings from this study will help inform present-day clinical reasoning and support the design and direction of future larger-scale primary studies examining lower-limb injury prevention in Spanish elite male and female youth soccer players.

## Figures and Tables

**Table 1:** Anthropometric Data.

Variable	Sex	Mean	SD	Median	IQR	ES
Age	M	16.8	0.9	17.0	2.0	0.0
	F	17.2	1.7	17.0	2.0	
Standing Height (cm)	M	175.9	6.8	176.6 *	10.3	0.7
	F	164.0	6.3	164.0	7.0	
Sitting Height (cm)	M	135.4	3.5	135.0 *	4.6	0.8
	F	127.8	3.5	127.0	5.0	
Body Mass (kg)	M	67.9	6.3	67.6 *	8.5	0.7
	F	59.0	5.8	58.3	8.5	
Right Leg Length (cm)	M	91.4	5.0	90.1 *	8.1	0.5
	F	86.6	4.5	87.0	4.0	
Left Leg Length (cm)	M	91.8	5.3	91.0 *	8.6	0.5
	F	86.8	4.5	87.0	5.0	

SD standard deviation; IQR interquartile range; ES effect size; M males; F females; * significant between-sex difference, *p* < 0.003.

**Table 2:** Balance and range-of-motion data.

Variable	Sex	Mean	SD	Median	IQR	ES
Right ART (cm)	M	67.7	9.9	66.0	9.0	0.2
	F	64.4	4.4	63.5	5.9	
Right %LL ART	M	74.0	10.0	72.0	10.0	0.1
	F	74.0	4.0	74.0	6.00	
Left ART (cm)	M	68.0	9.7	67.0	9.0	0.3
	F	63.9	6.6	63.8	4.4	
Left %LL ART	M	74.0	9.0	73.0	8.0	0.1
	F	73.0	6.0	74.0	7.0	
Right HIR (°)	M	35.5	9.9	35.0 *	10.0	0.6
	F	46.9	9.7	45.0	15.0	
Right HER (°)	M	65.7	9.9	70.0	10.0	0.1
	F	68.0	9.5	65.0	10.0	
Left HIR (°)	M	39.9	8.8	40.0 *	10.0	0.6
	F	51.1	9.9	55.0	10.0	
Left HER (°)	M	65.0	8.7	65.0	10.0	0.1
	F	64.4	12.5	65.0	15.0	
Right AKE test (°)	M	26.7	8.9	30.0	10.0	0.1
	F	24.9	8.5	25.0	10.0	
Left AKE test (°)	M	25.9	9.3	25.0	10.0	0.1
	F	27.7	11.0	30.0	15.0	
Right Dorsiflexion (°)	M	36.6	6.5	37.0	7.0	0.2
	F	39.2	5.0	40.0	8.0	
Left Dorsiflexion (°)	M	37.9	7.4	39.0	8.0	0.1
	F	39.9	4.6	39.0	6.0	

SD standard deviation; IQR interquartile range; ES effect size; ART anterior reach test; %LL percentage of leg length; HIR hip internal rotation; HER hip external rotation; AKE active knee extension; M males; F females; * significant between-sex difference, *p* < 0.003.

## Data Availability

The data presented in this study are available on request from the corresponding author.
